# Safety and efficacy of the combination of erlotinib and sirolimus for the treatment of metastatic renal cell carcinoma after failure of sunitinib or sorafenib

**DOI:** 10.1038/sj.bjc.6605868

**Published:** 2010-09-07

**Authors:** T W Flaig, L J Costa, D L Gustafson, K Breaker, M K Schultz, F Crighton, F J Kim, H Drabkin

**Affiliations:** 1 Division of Medical Oncology, Department of Medicine, University of Colorado Denver School of Medicine, Mail Stop 8117, 12801 East 17th Avenue Room 8117, Aurora, CO 80045, USA; 2 Division of Hematology-Oncology, Department of Medicine, Medical University of South Carolina, Charleston, SC, USA; 3 Cancer Center Pharmacology Core, Colorado State University, Fort Collins, CO, USA; 4 University of Colorado Hospital, Aurora, CO, USA; 5 Division of Urology, Department of Surgery, University of Colorado Denver School of Medicine, Aurora, CO, USA; 6 Division of Urology, Department of Surgery, Denver Health Medical Center, Denver, CO, USA

**Keywords:** carcinoma, renal cell, receptor, epidermal growth factor, sirolimus, drug therapy, combination

## Abstract

**Background:**

The mammalian target of rapamycin (mTOR) is an important therapeutic target in the treatment of renal cell carcinoma (RCC). Pre-clinical data indicate that the combined inhibition of both the epidermal growth factor receptor and mTOR results in enhanced anticancer activity.

**Methods:**

All patients had metastatic RCC with progression after treatment with sunitinib and/or sorafenib. Treatment consisted of erlotinib 150 mg orally once a day starting on day 1 and sirolimus 6 mg orally on day 8 followed by 2 mg daily, adjusted according to blood levels.

**Results:**

A total of 25 patients were enrolled between July 2006 and March 2008. The median progression-free survival (PFS) was 12 weeks (95% CI 5.9–18.1) and median overall survival (OS) 40 weeks (95% CI 0–85.7). No confirmed complete or partial responses were observed, but stable disease >6 months was noted in 21.8% (95% CI 4.9–38.6) of patients. The most common adverse events were rash and diarrhoea. There was no correlation between erlotinib, OSI-420 (days 8 and 15) or sirolimus (days 15 and 29) blood levels and PFS or OS.

**Conclusions:**

The combination of sirolimus and erlotinib for RCC failed to demonstrate an advantage over available single-agent therapy in the second-line setting.

Renal cell carcinoma (RCC) affects near 58 000 individuals in the United States every year, and metastatic disease causes nearly 13 000 deaths annually (Jemal *et al*, 2009). With the exception of a small proportion of selected patients treated with high-dose interleukin-2 (McDermott *et al*, 2005) or allogeneic hematopoietic stem cell transplantation (Childs *et al*, 2000), metastatic RCC remains an incurable condition.

In the majority of cases of RCC, functional inactivation of the von Hippel–Lindau (*VHL*) gene leads to overexpression of hypoxia inducible factor (HIF)-1*α* and HIF-2*α* resulting in overproduction of vascular endothelial growth factor (VEGF) and transforming growth factor (TGF)-*α* (Kim and Kaelin, 2006). The development of oral inhibitors of the kinase activity of the VEGF receptors, namely sunitinib (Motzer *et al*, 2007) and sorafenib (Escudier *et al*, 2007), represent a major therapeutic advancement in the treatment of RCC. The best response to multi-kinase inhibitors is often temporary disease stabilisation, although long-term clinical response is seen in a small number of patients.

The mammalian target of rapamycin (mTOR) is a serine–threonine kinase implicated in cellular growth and proliferation. It is regulated directly or indirectly by growth factor receptors and cell signalling pathways known to be overstimulated in RCC, particularly the PI3 kinase pathway (Guertin and Sabatini, 2007). Two analogues of sirolimus (rapamycin), temsirolimus (Hudes *et al*, 2007) and everolimus (Motzer *et al*, 2008), have shown clinical activity in metastatic RCC as initial treatment of high-risk disease and after failure of a multi-kinase inhibitor, respectively.

As TGF-*α* overproduction and consequent stimulation of the epidermal growth factor receptor (EGFR) is a hallmark of most RCC tumours (Gunaratnam *et al*, 2003; Gemmill *et al*, 2005; Costa *et al*, 2007), EGFR inhibitors (such as erlotinib and gefitinib) have a theoretical role in the treatment of RCC, on the basis of our understanding of the pathophysiology. In previous work, our group has demonstrated *in vitro* synergistic activity between mTOR inhibitors and EGFR inhibitors at concentrations that are achievable *in vivo*, providing the rationale for this clinical study (Gemmill *et al*, 2005; Costa *et al*, 2007).

In this study, we provide the mature results of a single-institution phase II trial examining the clinical activity of the combination of sirolimus and erlotinib in a pretreated population of RCC patients.

## Materials and methods

This was a phase II, single-arm, single-institution trial. The primary objective was to evaluate the efficacy of the combination of sirolimus and erlotinib as determined by progression-free survival (PFS). Secondary objectives were evaluation of the safety and tolerability of the combination, response rate, overall survival (OS) and the influence of sirolimus on the blood levels of erlotinib as well as correlation between drug levels and antitumour activity.

### Patients

All patients had a histological diagnosis of RCC, life expectancy of >3 months, an ECOG performance status of 0–2, measurable disease, progression after or intolerance to sorafenib and/or sunitinib, and adequate bone marrow function (haemoglobin of ⩾9 g per 100 ml, platelets of ⩾100 000 × 10^9^ and an absolute neutrophil count of ⩾1500 mm^3^). The exclusionary criteria included previous treatment with erlotinib, gefitinib, sirolimus, temsirolimus or everolimus, untreated central nervous system metastasis, renal failure requiring dialysis or significant liver dysfunction (AST, ALT, total bilirubin or alkaline phosphatase >1.5 the upper limits of normal). The protocol was approved by the Colorado multiple institutional review board and all participants provided written informed consent before study enrollment. The study was registered on ClinicalTrials.gov (Identifier: NCT00353301).

### Treatment

Erlotinib (Tarceva, Genentech USA, South San Francisco, CA, USA) therapy was started at 150 mg by mouth daily from day 1 to be taken at least 1 h before or 2 h after a meal. Sirolimus was initiated on day 8 (D8) with a loading dose of 6 mg by mouth, followed by a daily dose of 2 mg by mouth, on the basis of the product prescribing information for low-to-moderate immunologic risk renal transplant patients. The levels of erlotinib and its primary metabolite, OSI-420, were assessed after the initial 7 day run-in and after 1 week of combined therapy (i.e., on days 8 and 15). Erlotinib levels were not used to make clinical decisions. Trough levels of sirolimus were checked on day 15 (D15) and then monthly and maintained between 4 and 20 ng ml^–1^. The sirolimus dose was also reduced because of any significant toxicity attributed to sirolimus. Treatment was continued until progression, unacceptable toxicity, withdrawal of consent or investigator's decision to discontinue therapy.

All participants had measurable disease with repeated computed tomography or magnetic resonance imaging scans every 8 weeks while on the study. A bone scan was assessed at baseline and followed in those with bone disease at baseline or in any patient with new bone symptoms. Physical exams, toxicity assessments and laboratory assessments (including electrolytes, kidney function, liver function, complete blood count and fasting lipids) were obtained every 4 weeks.

Progression was defined as radiographic progression according to RECIST criteria (year 2000 version), non-compliance in obtaining scans, unequivocal clinical progression or the initiation of another medication for the treatment of RCC. The safety of this combination was assessed through the compilation and grading of all adverse events (AEs) according to the common terminology criteria for AEs v3.0.

### Erlotinib and OSI-420 analysis in human plasma by LC/MS/MS

Erlotinib and OSI-420 were measured in human plasma using a validated LC/MS/MS assay based on a previously published method with midazolam as an internal standard (Zhao *et al*, 2003). OSI-420 is an active metabolite of erlotinib. Positive ion electrospray ionisation mass spectra were obtained with a MDS Sciex 3200 Q-TRAP triple quadrupole mass spectrometer (Applied Biosystems, Inc., Foster City, CA, USA) with a turbo ionspray source interfaced to an Agilent 1200 Series Binary Pump SL HPLC system (Santa Clara, CA, USA). Samples were chromatographed with an XBridge Phenyl, 2.5 *μ*m, 4.6 × 50 mm column (Waters Corporation, Milford, MA, USA) protected by a C18 guard cartridge, 4.0 × 2.0 mm (Phenomenex, Torrance, CA, USA). An LC gradient was used with mobile phase A consisting of 10 mM ammonium acetate and mobile phase B consisting of acetonitrile. Chromatographic separation was achieved by increasing mobile phase B linearly from 30 to 98% from 0 to 1.75 min, maintaining at 98% from 1.75 to 2.25 min, decreasing linearly from 98 to 30% from 2.25 to 2.5 min, followed by re-equilibration of the column at 30% mobile phase B from 2.5 to 3 min. The LC flow rate was 1.3 ml min^–1^, the sample injection volume was 5 *μ*l and the analysis run time was 3 min.

The mass spectrometer settings were optimised as follows: turbo ionspray temperature, 650°C; ion spray voltage, 2000 V; declustering potential, 50 V; entrance potential (EP), 10 V; collision energy, 45 V (Erlotinib and OSI-420) and 39 V (midazolam); collision cell EP, 150 V (Erlotinib and OSI-420) and 35 V (midazolam); collision cell exit potential, 5 V (Erlotinib and midazolam) and 10 V (OSI-420); curtain gas, N_2_, (CUR), 30 units; collision gas, N_2_, (CAD), medium; nebuliser gas, N_2_, 60 units; and auxiliary gas, N_2_, 60 units. Samples were quantified by internal standard reference method in the MRM mode monitoring ion transitions *m/z* 394 → 278 a.m.u. for erlotinib, *m/z* 380 → 278 a.m.u. for OSI-420 and *m/z* 326 → 291 a.m.u. for the internal standard, midazolam. Each ion transition was integrated for 200 ms and Q_1_ and Q_3_ were both operated in unit resolution mode.

Analytical standards, quality control (QC) and unknowns were all prepared by adding 200 *μ*l of unknown or spiked blank plasma samples to a 1.5 ml microcentrifuge tube containing 10 *μ*l of 10 *μ*g ml^–1^ midazolam solution followed by brief vortexing. Plasma proteins were then precipitated by the addition of 800 *μ*l of acetonitrile followed by 10-min vortex mixing. Samples were then centrifuged at 18 000 RCF for 10 min and the supernatant collected and transferred to autosampler vials for analysis. Erlotinib, OSI-420 and midazolam standard solutions were prepared in 50% acetonitrile in water. The lower and upper limits of quantitiation for the assay were 0.5 and 5000 ng ml^–1^, respectively. Accuracy and precision (% RSD) based on analysis of QC samples for this assay was 96.5 and 3.4% for erlotinib and 96.8 and 1.9% for OSI-420.

### Statistical analysis

A sample size of 25 patients was calculated to provide 80% power to detect an improvement in PFS from 12 to 24 weeks in this population. Progression-free survival and OS with their respective 95% CI were calculated using the method of Kaplan–Meier. Comparison between drug levels on days 8 and 15 was performed using Wilcoxon signed-rank test. Drug levels between patients who had stable disease (SD) and patients who had progressive disease as their best response were compared using Mann–Whitney *U-*test. All statistical inferences were based on a *P*-value of <0.05.

## Results

### Patient characteristics

Enrollment started in July 2006 with the last patient accrued in March 2008. A total of 27 patients were screened of which 25 were found to be eligible and continued to treatment. The median subject age was 60 years (range of 47–73 years), with an average of 2.6 previous medical treatments for RCC. All patients had histologically confirmed RCC with 19 having predominantly clear cell type (Table 1). Data were gathered and analysed in October 2009 with the median follow-up of surviving patients being 117 weeks.

### Efficacy

**Figure 1:**
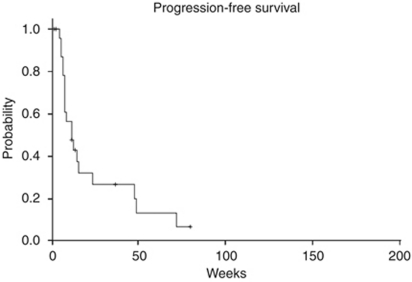
Kaplan–Meier estimate of the PFS.

**Figure 2:**
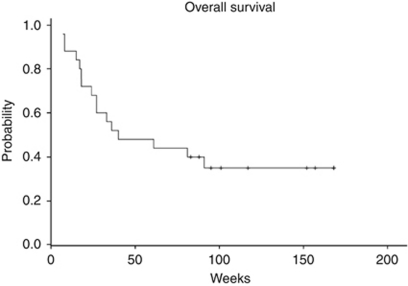
Kaplan–Meier estimate of the OS.

Two patients withdrew consent before first response assessment or obvious progression. These patients are assessed for toxicity but not for efficacy. The median PFS was 12 weeks (95% CI 5.9–18.1) as displayed in Figure 1. The median OS was 40 weeks (95% CI 0–85.7, Figure 2). There were no objective responses according to RECIST criteria; however, 13 patients had SD beyond 8 weeks (56.5%, 95% CI 36.3–76.8) and of these, 5 (21.8, 95% CI 4.9–38.6) had SD lasting longer than 6 months. One subject had SD lasting 80+ weeks and was still on therapy at the time of this report. An analysis of best response by RECIST criteria indicates that 7 of 25 patients had a reduction in their RECIST measurements from baseline, ranging from a 1.8 to 17.0% reduction.

**Figure 3:**
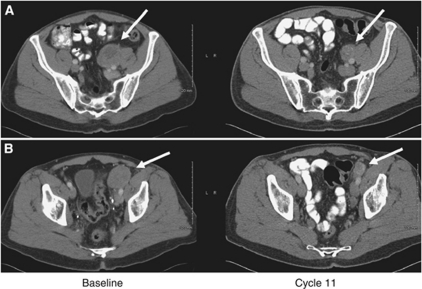
(**A**) Evidence of tumour response. Soft tissue pelvic mass measuring 55 mm at baseline (left panel) *vs* 30 mm at cycle 11 (right panel); (**B**) a second pelvic mass in the same patient, 50 mm at baseline and 34 mm at cycle 11. Other target lesions were stable and this subject did not meet RECIST criteria for a partial response.

Several patients had notable reduction in the size of some tumours, although none of these reductions met the RECIST criteria for a partial response. Figure 3 shows an example of a significant reduction in the size of two pelvic tumours, although tumours in the patient's abdomen later grew during the course of his therapy, leading to progressive disease after completing 48 weeks with SD.

### Toxicity

Frequent and severe AEs are displayed in Table 2. The most common AE observed was rash, consistent with the rash associated with single-agent erlotinib use and observed in 24 patients. It was generally mild-to-moderate (19 of 24 cases were grade 1 or 2) and centred on the face and upper thorax. Diarrhoea was also frequently encountered, observed in 12 subjects, although it was grade 1 in 10 of the 12. Special attention was paid to hypertriglyceridaemia, as this is a known side effect of sirolimus. It was noted in 9 of the 25 subjects, without clear clinical symptoms (e.g., pancreatitis) in any participant. Overall, the most common grade 3 and 4 toxicities (at least 2 incidences) included: rash, anaemia, mucositis, hypoalbuminaemia, and hypophosphataemia. Two subjects died during the study period. One patient discontinued therapy and died of disease progression during the 30-day safety window. Another patient stopped treatment 7 days before a planned tumour debulking and died in the postoperative period.

### Drug levels and efficacy

The levels of erlotinib and its active metabolite OSI-420 were assessed on D8 and on D15 of cycle 1. D8 values were available for 24 subjects and D15 values for 22 subjects. The introduction of sirolimus on D8 did not cause any significant change in blood levels of erlotinib (*P*=0.54) or in the levels of OSI-420 (*P*=0.60), comparing D8–D15. Neither D8 erlotinib (*P*=0.72), D15 erlotinib (*P*=0.40), D8 OSI-420 (*P*=0.48), D15 OSI-420 (0.89), D15 sirolimus (*P*=0.99) nor D29 sirolimus (*P*=0.25) correlated with PFS. Similarly, neither D8 erlotinib (*P*=0.23), D15 erlotinib (*P*=0.11), D8 OSI-420 (*P*=0.44) D15 OSI-420 (*P*=0.17), D15 sirolimus (*P*=0.64) nor D29 sirolimus (*P*=0.48) correlated with the OS. An analysis of the erlotinib, OSI-420 and sirolimus drug levels with respect to the best response to therapy, showed no correlation (Table 3).

## Discussion

Since the initiation of this study, two mTOR inhibitors have been approved by the US Food and Drug Administration for the treatment of RCC (Hudes *et al*, 2007; Motzer *et al*, 2008). Temsirolimus and interferon were evaluated in the first-line RCC setting, specifically including patients with poor clinical prognostic factors (Hudes *et al*, 2007). Single-agent temsirolimus demonstrated a 3.5-month median survival advantage (7.3 *vs* 10.9 months), compared with interferon. More recently, an oral mTOR inhibitor, everolimus, was compared with placebo in the treatment of RCC after failure of a tyrosine kinase inhibitor (Motzer *et al*, 2008). The final results of this trial have now been released, showing a persistent benefit from everolimus as measured by PFS (4.9 *vs* 1.9 months), but not by OS (Motzer *et al*, 2010). The objective response rate in the final analysis was 1.8% with everolimus *vs* 0% with placebo, although 47% of everolimus-treated patients had some reduction in tumour size from baseline, *vs* only 10% of subjects in the placebo arm. In this study, 7 of 25 (28%) of the patients had some reduction in tumour, by RECIST measurements. Unlike the multi-targeted tyrosine kinase inhibitors, such as sunitinib and sorafenib, which target a complex array of different enzymes, sirolimus, temsirolimus and everolimus share a single target (mTOR) and are closely related in structure and activity. It remains unclear what advantages any of these individual agents may possess over the others in this class.

This study demonstrates the safety and feasibility of treatment combining EGFR and mTOR inhibition in a pretreated cohort of advanced RCC patients. We were unable to prove a median PFS superior to 12 weeks, the main hypothesis stated in the study design. Indeed, a median PFS of 1.9 months was seen in the placebo-treated control arm of a trial accruing a similar population of RCC patients (Motzer *et al*, 2008). This suggests that we may have failed to reject the null hypothesis because we overestimated the PFS in absence of treatment rather than a lack of activity of the study combination. However, a proportion of patients in the current trial obtained prolonged SD, 21.8% at 6 months, which compares with approximately 35 and 10% in the everolimus and placebo arms of the phase III trial at 6 months, respectively (Motzer *et al*, 2010).

The clinical significance of EGFR inhibition in RCC remains uncertain. Several lines of evidence including a strong mechanistic rationale and preclinical data support the importance of the EGFR pathway in RCC. Specific work directly endorses a synergistic role for EGFR and mTOR inhibition in RCC cells *in vitro* (Gemmill *et al*, 2005; Costa *et al*, 2007). There are now several completed trials examining EGFR blockade in RCC. The activity of gefitinib as a single-agent has been evaluated in RCC patients with metastatic disease with no objective responses observed (Jermann *et al*, 2006). The median time to progression was 110 days, but the significance of this finding is difficult to assess without a control arm. A separate investigation of single-agent gefitinib in advanced RCC sought to correlate EGFR protein expression to clinical response (Dawson *et al*, 2004). A total of 21 patients were treated without any objective responses, yielding a median PFS of 2.7 months. There was no correlation between EGFR status and the PFS. In contrast, lapatinib, a dual-EGFR/ErbB2 inhibitor, was shown to improve OS specifically in the subset of RCC patients with elevated EGFR expression (Ravaud *et al*, 2008). EGFR inhibitors have also been combined with other anti-vascular agents. Bukowski *et al* (2007) randomised 104 patients with RCC to bevacizumab with or without erlotinib in the first-line setting. Although well tolerated, the combination treatment did not yield any improvement in PFS over bevacizumab alone. Thus, on the basis of our results and these studies, we suggest that future investigation of EGFR inhibitors in RCC should focus on the subset of tumours that overexpress (or have mutated) ERB-B receptors. As demonstrated in clinical studies of EGFR inhibitors in lung cancer, the benefit of EGFR-targeted therapies may not be apparent in unselected patients (Herbst *et al*, 2004), while marker-positive patients may have significant benefit (Maemondo *et al*, 2010).

Recent studies have demonstrated that while rapamycin effectively inhibits mTORC1-mediated phosphorylation of p70S6 kinase and its substrates, inhibition of 4E-BP1 in mammalian cells is frequently deficient (Thoreen *et al*, 2009). This is in distinct contrast to rapamycin-mediated Tor inhibition in yeast. Therefore, it is likely that the degree of mTOR inhibition obtained with the current agents in human cancers, including RCC, is insufficient.

With the recent approval of six targeted agents for the treatment of RCC, clinicians now face a therapeutic dilemma in selecting the most appropriate agent for an individual RCC patient. Unlike other cancer types in which predictive biomarkers exist for targeted therapies (e.g., HER2/neu in breast cancer), therapeutic decision-making in RCC relies on rudimentary and largely clinical measures including performance status, past treatments and histology. To make the best use of our current therapeutic tools, new predictive biomarkers must be validated in RCC in carefully designed trials able to discriminate between prognostic and predictive markers. In this arena, the VHL mutational status has been correlated in the preclinical setting to responses with mTOR and EGFR inhibition, with wt-VHL demonstrating enhanced activity (Gemmill *et al*, 2005). One limitation of this study is the lack of VHL mutational status assessment. It is also notable that there is very limited evidence suggesting that activating EGFR mutations may be present in RCC and predict for EGFR tyrosine kinase inhibition responses (Iyevleva *et al*, 2009). Such mutations were not assessed in this study.

In summary, the combination of erlotinib and sirolimus has an acceptable safety profile in RCC, considering the advanced disease state of the subjects in this trial. Although no objective responses were noted, prolonged SD was observed in a small number of patients; however, the combination failed to demonstrate an advantage over single-agent everolimus in this setting. The clinical importance of EGFR blockade and the relative effectiveness of any one of the available mTOR inhibitors over another in RCC remain uncertain, although the use of sirolimus would represent a considerable cost savings.

## Figures and Tables

**Table 1:** Patient characteristics

Number of treated patients	25
*Median age*	60 years
Age range	47–73 years
Mean number of previous treatments	2.6
Previous treatment number range	1–6
Previous sunitinib treatment	22
Previous sorafenib treatment	19
Previous bevacizumab treatment	5
*Histology distribution*	
Clear cell carcinoma (predominant)	19
Clear cell with sarcomatoid features	3
Unclassified/other renal cell carcinoma	3

**Table 2:** Adverse events

**Symptoms**	**Grade 1**	**Grade 2**	**Grade 3**	**Grade 4**	**Total**
*Dermatology/skin*					
Rash	16	3	5		24
Dry skin	7	1			8
Pruritis/pain	4	2			6
					
*Haematologic/lab*					
Anaemia	2	7	5		14
					
*GI*					
Diarrhoea	10	1	1		12
Anorexia	7	2			9
Mucositis	4	3	2		9
Nausea	7	1			8
					
*Metabolic/lab*					
Hypoalbuminaemia	9		3		12
Elevated creatinine	6	3	1		10
Hypertriglyceridaemia	2	6	1		9
Hypophosphataemia	2	3	2		7
					
*Constitutional*					
Fatigue	9	2			11
Pain	5	2			7
Chills/cold intolerance	6				6
Weight loss	6				6
					
*Pulmonary*					
Cough	7				7
Rhinitis	7				7
Pulmonary oedema			1		1
					
*Haemorrhage/bleeding*					
Haemorrhage/bleeding other	6				6
Epistaxis	6				6
					
*Cardiac*					
Decreased LVEF			1		1
MI			1		1
Tachycardia			1		1
Anuria			1		1

Abbreviations: GI=Gastrointestinal; LVEF=Left ventricular ejection fraction; MI=myocardial infarction. All serious adverse events or adverse effects experienced by six or more patients are listed above with or without attribution to study medications.

**Table 3:** Drug and metabolite levels according to best response

	**Stable disease (ng** **ml^–1^)**	**Progressive disease (ng** **ml^–1^)**	***P*-value**
D8 erlotinib	1792	2201	0.2
D15 erlotinib	1853.5	1652.5	0.74
D8 OSI-420	154.5	193	0.18
D15 OSI-420	155.5	131	0.91
D15 sirolimus	11.5	9.5	0.65
D29 sirolimus	7.0	8.1	0.46

Abbreviations: D8=day 8 of cycle 1; D15=day 15 of cycle 1; D29=day 29 of cycle 1.

It is to be noted that two subjects withdrew consent before the first imaging assessment or progression and are not included; therefore the total number of subjects analysed in this assessment is 23.
